# Frailty and functional dependence in older population: lessons from the FREEDOM Limousin – Nouvelle Aquitaine Cohort Study

**DOI:** 10.1186/s12877-022-02834-w

**Published:** 2022-02-14

**Authors:** Sophie Boyer, Justine Trimouillas, Noëlle Cardinaud, Caroline Gayot, Cécile Laubarie-Mouret, Nathalie Dumoitier, Karen Rudelle, Michel Druet-Cabanac, Marie-Laure Laroche, Achille Tchalla

**Affiliations:** 1 Laboratoire VieSanté - UR 24134 (Vieillissement, Fragilité, Prévention, e-Santé), Institut Ω-Health, Université de Limoges, Limoges, France; 2 UPSAV (Unité de Prévention, de Suivi et d’Analyse du Vieillissement), Pôle HU Gérontologie Clinique, CHU de Limoges, 2 Avenue Martin-Luther, F-87042 Limoges, King, France; 3 Unité de Recherche Clinique et d’Innovation (URCI) de Gérontologie, Pôle HU Gérontologie Clinique, CHU de Limoges, Limoges, France; 4 Département de Médecine Générale, Faculté de Médecine de Limoges, Limoges, France; 5 Centre de Pharmacovigilance et de Pharmacoépidémiologie, CHU de Limoges, Limoges, France; 6 Geriatric Medicine Department, University Hospital Centre, 2 Avenue Martin Luther King, 87042 Limoges, France

**Keywords:** Aging, autonomy, disability, elderly, frailty, independence

## Abstract

**Background:**

Monitoring frailty indicators in elderly people is recommended to identify those who could benefit from disability prevention programs. To contribute to the understanding of the development of frailty in the elderly, we have created the FREEDOM-LNA cohort constituting an observational study of ageing in general population. Here, we described the characteristics of a cohort of elderly subjects who are followed for determination of frailty and loss of independence trajectories.

**Results:**

The cohort was composed of 1085 subjects in advanced age (mean: 83.7 ± 6.0 years) and of women in majority (68.3%). Cardiovascular risk factors were present in 88.4% of subjects. Abnormal musculoskeletal signs were reported in 44.0% and neurologic signs in 31.9%. There were 44.8% of subjects at risk of malnutrition (MNA <24) and 73.3% (668/911) at risk of mobility-related disability (SPPB ≤9); 39% (384/973) of subjects had impaired cognitive function (MMSE< 24, adjusted on education) and 49.0% (397/810) had signs of depression (GDS >9); 31.8% (240/753) were frail and 58.3% were pre-frail. Most subjects had at least one disability in ADL (66.9%) and IADL (85.1%). The SMAF indicated a loss of independence in 59.6%. Overall, 59.9% of subjects could not stay at home without at least some help. Consequently, a medical consultation was proposed in 68.2 and 42.1% social supports.

**Conclusions:**

A large part of this cohort was frail or pre-frail and presented signs of loss of independence, which may be explained by multiple factors including impaired health status, poor physical performance, cognition, isolation, depression, or nutrition. This cohort will help to determine factors that adversely influence the trajectory of physical frailty over time.

**Supplementary Information:**

The online version contains supplementary material available at 10.1186/s12877-022-02834-w.

## Introduction

The rise in life expectancy is one of the most remarkable advances of the last century around the world. The increased longevity is however challenged by the ageing population especially in developed countries [1, 2]. In Europe, 24% of the population is aged over 60 years and, with the post-war baby-boom generation, that proportion is projected to reach 34% in 2050. [2] Longer life promotes a progressively higher prevalence of chronic age-related comorbidities and disabling illness, including cardiovascular, metabolic, musculoskeletal, sensorial and cognitive disorders, and increasing risk of psychological distress, social disconnection, loss of independence, and dependency at the end of life [3, 4]. In line with the geriatric community, [5–7] the World Health Organization (WHO) recently asked to adopt a global strategy to keep the elderly healthy, including providing long-term integrated care to maintain a level of functional ability in an age-friendly environment [4]. The objective is to keep people healthy based on the notion of functional ability and not just to treat the acute or chronic diseases [4, 5, 8].

Pathological aging as opposed to healthy aging occurs when the organism at various organ levels is unable to compensate for age and disease-related changes [9]. On the other hand, physical and functional decline may occur in the absence of identifiable disease which has led to the concept of frailty. Frailty is defined as an age-related state of decline and vulnerability characterized by decreased physiological reserves and function across multiple organ systems. Frail people are less resilient to sudden changes in health status even minor stressor such as mild acute illness or physical or psychological trauma, and are thus at increased risk of adverse aged-related outcomes such as falls, hospitalizations, disability and morbi-mortality [10, 11]. There is a considerable overlap between comorbidity, frailty and disability [12, 13]. Contrary to disability, there is current consensus that frailty is potentially reversible with appropriate interventions including physical activity, nutrition, and cognitive training in older adults. [14] Thus, monitoring frailty indicators in community-dwelling elderly people is recommended to identify old people who could benefit from disability prevention programs [15–18]. Research is also needed to determine how physical, psychological, and social conditions are associated with frailty and functional status and to determine factors that adversely influence the trajectory of physical frailty over time [19].

To contribute to the understanding of the development of frailty in the elderly, we have created the FREEDOM-LNA cohort (French acronym for Frailty, Clinical Research and Evaluation at Home in Limousin – Nouvelle Aquitaine) constituting an observatory of ageing in general population. We performed prospective and retrospective analyses of frailty, functional loss, and cognition in community-dwelling elderly with the objective to determine factors associated with frailty trajectories. A secondary objective was to analyse the different trajectories of loss of independence. In this preliminary report, we described the profile of this cohort population including health and socio-environmental factors, the loss of functional independence, and the appropriate geriatric interventions proposed to stay longer at home.

## Materials and methods

### Study design

FREEDOM-LNA was an historical longitudinal cohort conducted by the UPSAV (University Hospital, Clinical Geriatric Department, Limoges, France). The UPSAV is a clinical unit composed of a dedicated multidisciplinary team of geriatric physicians, nurses, ergotherapists, psychomotor therapists, and social workers. The team provides global preventive geriatric assessments in general population at home with the aim to detect the risk of loss of independence and the warning signs of frailty. Subjects are solicited from various information channels including healthcare professionals (e.g. family physicians, specialists, or hospitals), social professionals, closed relatives (family members or friends), or by the subject him/herself. The FREEDOM-LNA cohort comprised subjects aged ≥ 65 years with at least two comorbidities, or aged ≥ 75 years followed by the UPSAV between 01 January 2010 and 31 August 2017. All subjects were involved in a health care program that offered a comprehensive geriatric assessment every 6 months the first year and thereafter once a year. At the end of each assessment, the medical staff offered appropriate geriatric interventions including hygiene therapeutic advices, occupational therapist, psychomotor therapist, or social worker.

 The study protocol was reviewed and approved by the local Institutional Review Board (CEREES, Limoges; Approval number: TPS 429,669). The protocol was also approved by the French Data Protection Authority (CNIL) insuring protection of individualized data according to the French law. Informed consent for data processing was obtained from all subjects (or legal representatives). All procedures were carried out in accordance with the 1964 Helsinki Declaration and its later amendments.

### Measurements

#### Demographic, socio-environmental and clinical data

Demographic and socio-environmental characteristics were collected at inclusion and each follow-up visit. Self-reported supports including household incomes and financial supports, human supports and socio-medical supports and technical helps were also recorded using a specific questionnaire. A physical examination was performed and other clinical data, including medications, were obtained from self-reported questionnaire and from biological reports when available.

#### Nutritional status

The nutritional status was assessed using the Mini Nutritional Assessment (MNA). The full MNA includes 18 items grouped in 4 categories: anthropometric assessment; general assessment; short dietary assessment; and subjective assessment (self-perception of health and nutrition). Malnutrition was defined by a score < 17 and a risk of malnutrition by a score between 17 and 23.5 [20].

#### Physical activity and mobility

Mobility was assessed using the Short Physical Performance Battery (SPPB) which consists of a 4-meter walk at usual pace, a timed repeated chair stand, and three increasingly more difficult standing balance tests [21]. The total score ranges from 0 (worst) to 12 (best). A SPPB score ≤ 9 was suggesting for a risk of mobility-related disability.

#### Frailty

Frailty was assessed using the five phenotypic criteria as described by Fried et al. [10]: weakness as measured by grip strength (dominant hand < 20%), slowness (walking speed < 20% of normal), low level of physical activity in the last 2 weeks (<20% of energy expenditure, based on a physical activity questionnaire), low energy or self-reported exhaustion, and unintentional weight loss (4 to 5 kg since the previous year). Subjects were considered as frail when at least 3 criteria were present, pre-frail when there was one or two criteria and robust when there was no criteria.

#### Health status

The health status was assessed using the EuroQol-5 Dimension (EQ-5D). Each item of five dimensions (mobility, self-care, usual activities, pain/discomfort, and anxiety/depression) was scored using a 3-point scale (no problem=1, with problems=2; with extreme problems=3). The subjects were also asked to value their own health status on an analogue scale (EQ-VAS) ranging from 0 (the worst possible health status) to 100 (the best possible health status) [22].

#### Cognitive and psychosocial status

Neurocognitive domains such as verbal memory, immediate memory, and executive functioning were assessed using various neuropsychological tests including the Mini Mental State Examination (MMSE) questionnaire (30 items, scored between 0 and 30), [23] the 5-word test (5WT), [24] the clock drawing test (CDT), [25] the Controlled Word Association Test, [26] and the Category Naming Test [27]. Subjects were considered to have a cognitive deficit if MMSE was ≤ 20 in subjects with low education, ≤23 in subjects with medium education and ≤26 in subjects with a high education. A poor memory performance was indicated by 5-WT score ≤ 9. Depression over the past week was monitored using the Geriatric Depression Scale (GDS, 30 items); scores ranging from 0 to 5 indicate normal mood; scores between 5 and 9 indicate a risk of depressive symptoms, and scores > 9 indicate severe depressive symptoms [28].

#### Functional status

The functional status was assessed using the Katz’s index for basic daily living (ADL), and using the Lawton’s scale for instrumental activities of daily living (IADL) [29, 30]. An ADL ≤ 5 indicates dependency for daily activity, and an IADL ≤ 7 dependency for instrumental daily activities. Independence was also assessed using the SMAF (French acronym for Functional Autonomy Measurement System) questionnaire [31]. The SMAF is a 29-item scale and measures functional ability in 5 areas: daily living activities (7 items), mobility (6 items), communication (3 items), mental functions (5 items) and domestic tasks (8 items). For each item, the disability was scored on a 5-point scale: 0 (independent), -0.5 (with difficulty), -1 (needs supervision), -2 (needs help), and -3 (dependent). The total scored from 0 to -87 and a score ≤ -16 was subjective of a loss of independence. The level of dependency was also assessed using the actual legal instrument for evaluating dependency in elderly in France (AGGIR) [32]. This is a 17-item questionnaire which covered relatively complex activities related to physical or domestic functions (walking, dressing, toileting, household cleaning …), cognitive or social functions (cooking, medication use, finances, leisure, etc.). Each activity is scored according to three levels of dependency. This leads to calculate 3 degrees of dependency: strong dependency (GIR1 or 2), moderate dependency (GIR3 and 4) and weak dependency (GIR 5 or 6).

#### Geriatric intervention

Geriatric interventions such as therapeutic-hygienic and preventive advices; treatment modifications; additional medical and social assessments; reeducation/readaptation in an occupational therapist; psychosocial readaptation in a psychotherapist were proposed at the end of each visit according to the subject’s need.

### Statistical analyses

For subjects included between 2010 and 31 January 2014, data were recorded on the subject’s file and then entered in the software dedicated to the study. For subjects included between 01 and 2014 and 31 August 2017, data were directly entered in the software system. Statistical analyses were performed using the SAS software, version 9.4 (SAS Institute, Cary, NC, USA). The statistical analysis focused on subjects characteristics at first visit (inclusion). Quantitative variables were described using means, standard deviations (SD), medians, quartiles, minimal, and maximal values and qualitative data were described using number of cases and percentages. Missing data were not replaced, and percentages were calculated without accounting for missing data, unless otherwise specified.

## Results

Overall, 1337 subjects were included; 250 (18.7%) had no data recorded, and 2 subjects refused their data to be analysed. Thus, the analysed population was composed of 1085 subjects. Main subjects’ characteristics are provided in Table 1. The cohort was mainly composed of elderly subjects of 80 years old or above (73.6% of subjects) and of women in majority (68.3%), with a low/medium educational level (61.9%), and living alone (53.8%). Most subjects (88.5%) had at least one cardiovascular risk factor and are exposed to polypharmacy, 83.4% taking 5 and more medications daily. Clinical examination showed abnormal cardiovascular signs in 82.2% of subjects, musculoskeletal signs in 44.0%, and neurologic signs in 31.9% of subjects.

**Table 1:** Socio-demographic and clinical characteristics

**Age**, mean ± SD, (*N*=1071)	83.7 ± 6.0
**Sex**, women, n/N (%)	740/1085 (68.3%)
**Marital status and children **(*N*=1083)	
Married/common low partner	467 (43.1%)
Widowed/divorced/unmarried	616 (56.9%)
At least one children	957 (88.4%)
Living alone	583 (53.8%)
**Socio-professional category**^**a**^(*N*=1031)	
Low	736 (71.4%)
Intermediate	180 (17.5%)
High	115 (11.2%)
**Education**^**b**^(*N*=1083)	
Low	203 (18.7%)
Medium	468 (43.2%)
High	412 (38.0%)
**Vital signs **(mean±SD)	
Systolic blood pressure (mmHg), *N*=1039	134.3 ± 16.8
Diastolic blood pressure (mmHg), *N*=1039	73.3 ± 10.0
Heart rate (bpm), *N*=1018	70.9 ± 9.7
Body mass index (kg/m^2^), *N*=1054	26.5 ± 5.2
**Abnormal clinical examination **(*N*=1074)	
Cardiovascular	882 (82.1%)
Musculoskeletal system	473 (44.0%)
Neurologic	343 (31.9%)
Skin	170 (15.8%)
Abdomen	140 (13.0%)
Oral health	134 (12.5%)
Pulmonary	100 (9.3%)
Hydration	51 (4.7%)
**Cardiovascular morbidities**, n/N (%)	
Hypertension	792/1054 (75.1%)
Dyslipidaemia	497 /1049 (47.4%)
Obesity	272/1047 (26.0%)
Diabetes	226/1048 (21.6%)
Smoking	140/1045 (13.4%)
Alcohol consumption	47/1050 (4.5%)
At least one comorbidity	933/1055 (88.4%)
At least two comorbidities	605/1055 (57.3%)
5 and more medications daily	846/1014 (83.4%)
**Health status and frailty**	
MNA total score, mean ± SD, (*N*=1062)	23.3 ± 4.1
< 17	80/1062 (7.5%)
17 – 24	396/1062 (37.3%)
≥ 24	586/1062 (55.2%)
SPPB total score, mean ± SD, (*N*=911)	6.7 ± 3.6
0 – 6	428/911 (47.0%)
7 – 9	240/911 (26.3%)
10-12	243/911 (26.7%)
Fried total score, mean ± SD, (*N*=753)	2.0 ± 1.2
Frail	240/753 (31.9%)
Pre-frail	439/753 (58.3%)
Robust	74/753 (9.8%)
EQ-VAS, mean ± SD, (*N*=792)	57.8 ± 18.2
**Mental function**	
MMSE total score, mean ± SD, (*N*=973)	24.2 ± 4.9
Pathologic MMSE	384/973 (39.5%)
5-WT, mean ± SD, (*N*=826)	9.1 ± 1.7
5-WT < 9	170/826 (20.6%)
CDT failure	417/823 (50.7%)
At least one pathologic verbal fluency	510/727 (70.2%)
Pathologic categorial fluency	451/727 (62.0%)
Pathologic literal fluency	227/727 (31.2%)
GDS, mean ± SD, (*N*=810)	9.9 ± 5.5
5 – 9 (risk of depression)	272/810 (33.6%)
9 (depression)	397/810 (49.0%)
**Functional status**	
ADL score, mean ± SD, (*N*=1070)	5.0 ± 1.2
ADL ≤ 5	716/1070 (66.9%)
IADL score, mean ± SD, (*N*=1069)	5.1 ± 2.3
IADL ≤ 7	910/1069 (85.1%)
SMAF total score, mean ± SD, (*N*=1063)	-21.6 ± 14.5
0 – -8 (independent)	197/1063 (18.5%)
-8 – -16 (moderate dependency)	232/1063 (21.8%)
≤ -16 (loss of independence)	634/1063 (59.6%)
GIR score, mean ± SD, (*N*=1053)	4.6 ± 1.3
GIR 1 or 2 (strong dependency)	66/1053 (6.3%)
GIR 3 or 4 (moderate dependency)	474/1053 (45.0%)
GIR 5 or 6 (mild dependency)	513/1053 (48.7%)

^a^Low (employee, worker, farmer, housewife/husband)**; **High (Manager, Executive manager, licensed professional), intermediate (other status)

^b^Low (can read, write, count); medium (primary certificate level); high (secondary school, high school, university)

### Nutritional status, physical activities and frailty

Overall, the mean MNA score was 23.3 ± 4.1 and a risk of malnutrition (MNA < 24) was shown in 44.8% (586/1062) of subjects. The mean SPPB total score was 6.7 ± 3.6, and 73.3% (668/911) subjects had a SPPB ≤9, thus a risk of mobility-related disability.

The frailty total score was determined in 754 subjects; 240 (31.8%) were frail, 439 (58.2%) were pre-frail and 75 (9.9%) were robust. The most frequent frailty phenotype was “weakness” in 771/934 (82.5%) subjects and “low level of physical activity” in 587/969 (58.7%) subjects, while “slowness” was found in 262/792 (33.1%) subjects, “exhaustion” in 211/929 (22.7%) subjects, and “weight loss” in 106/1070 (9.9%) subjects.

### Mental functions

Thirty-nine percent (384/974) of subjects had a cognitive deficit (low MMSE adjusting for education); 20.6% (170/826) had a 5-WT < 9 indicating poor memory performances; 50.7% (417/823) failed on the clock-drawing test indicating some executive dysfunction; and 70.1% (510/727) had at least one categorial or literal fluency considered as pathologic, thus suspecting cognitive impairment. Regarding the depression scale, 33.6% (272/810) had a GDS score between 5 and 9 indicating a risk of depression and 49.0% (397/810) had a GDS score > 9, clearly indicating a depression.

### Functional status

The mean EQ-VAS was 57.8 ± 18.2 indicating impaired health status on average. The most affected dimension was pain/discomfort (88.8% including 9.3% with extreme pain/discomfort). Anxiety/depression was the second most affected dimension (67.2% reported moderate or extreme anxiety/depression). Using this questionnaire, 61.8% reported having difficulties in doing usual activities, 52.5% in washing or dressing alone, and 57.2% in walking (See Supplementary Table S1).

**Fig. 1:**
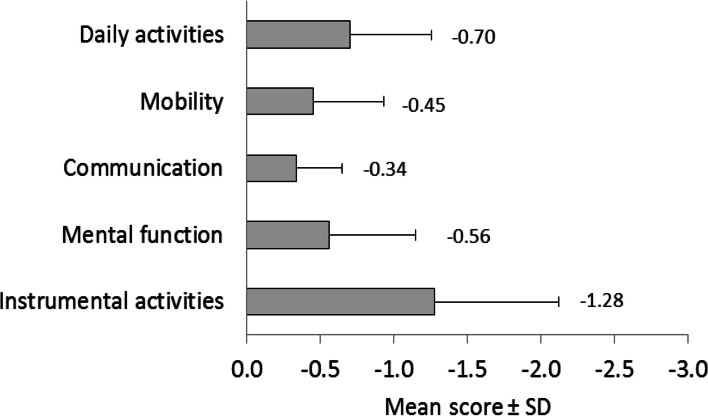
SMAF item for each domain. The mean ± SD score of each item of the 5 SMAF domains are indicated. A score of -1 indicates that subjects need some help to perform the function or activity, a score of -2 indicates a difficulty to perform the task, and a score of -3 the impossibility of doing the task

**Fig. 2:**
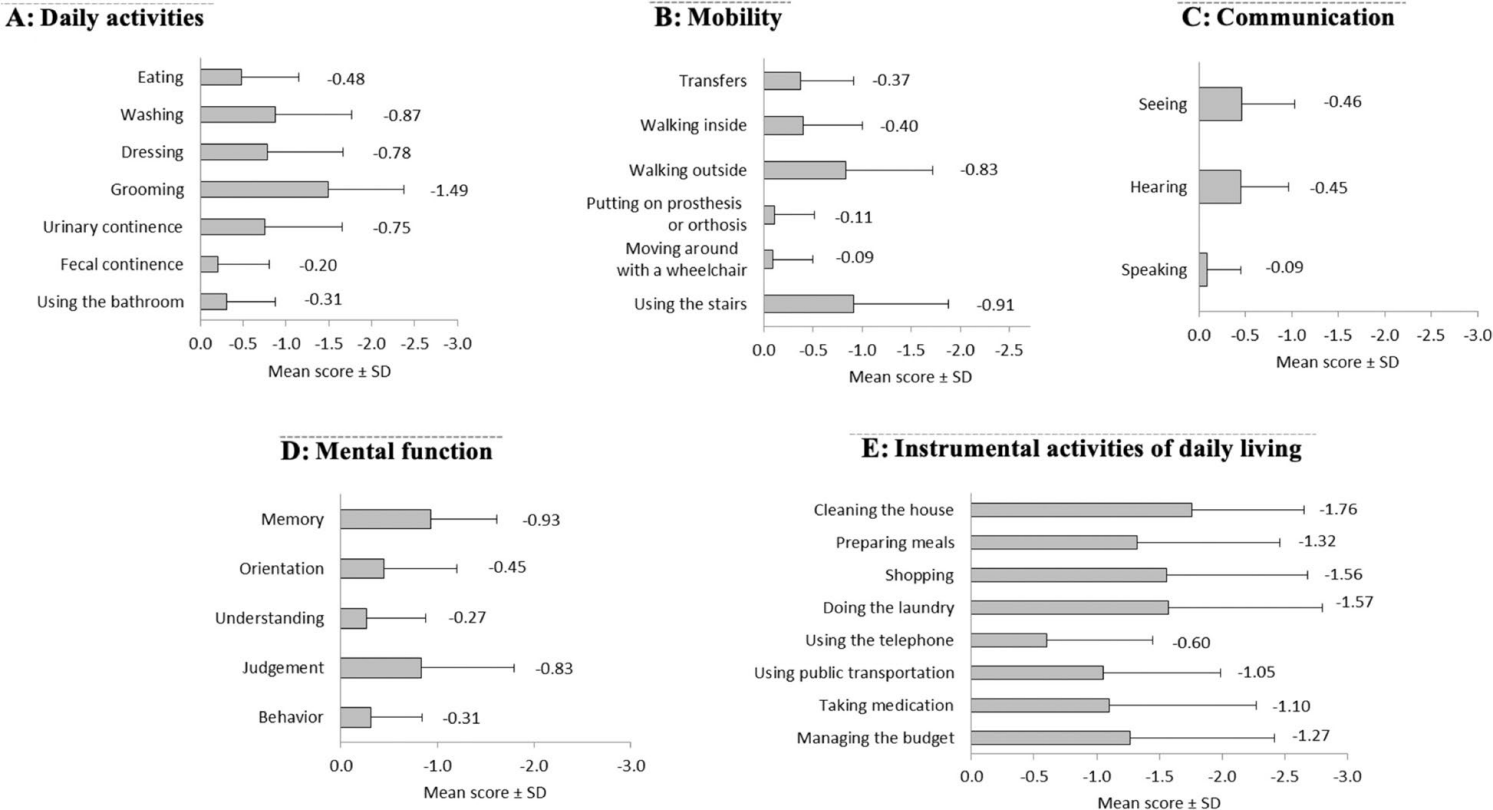
Score of each SMAF item. ** A** Daily activities; **B** Mobility; **C** Communication; **D** Mental function; **E** Instrumental activities of daily living

The mean GIR score was 4.6 ± 1.3 indicating that on average the cohort population had some dependency. Overall, 66.9% (716/1070) of subjects were dependent in ADL and 85.1% (910/1069) in IADL, and 59.6% (637/1063) had a SMAF ≤ -16 indicating a loss of independence. As shown in Fig. 1, the most affected SMAF dimension was “Instrumental activities of daily living” (mean score per item -1.28 ± 0.84) and then “Daily activities” (-0.70 ± 0.56). Beside difficulties in “Instrumental activities of daily living” (i.e. cleaning, cooking, shopping, laundry), the most severely affected activities was “grooming” (-1.49 ± 0.88); “using the stairs” (-0.91 ± 0.97), “walking outside” (-0.83 ± 0.89), memory (-0.93 ± 0.68) and judgement (-0.83 ± 0.96) (Fig. 2).

### Socio-environmental conditions and supports

A description of the main available supports including financial, human and technical is provided in Table 2. Most subjects (77.8%) were owner or had a free of charge lodging. The household income was quite low (< 1500 € per month) in 46.7% (485/1038). Almost all (97.2%) were covered by a private health insurance, and 73.1% for long-lasting illness. In addition, 31.3% received a personalised allowance of autonomy (i.e. monthly amount of 138 ± 243 €). Overall 89.8% of subjects could rely on human support including relatives (64.0%), nurses or home care nurse services (63.3%) and domestic help (52.9%). Regarding technical support, 83.8% used at least a technical help, mainly an alarm system (61.3%), grab bars (45.6%), or sticks (43.1%).

**Table 2:** Resources and supports

Lodging	N	1083
	Owner	646 (59.6%)
	Tenant	241 (22.3%)
	Usufruct/free of charge lodging	196 (18.1%)
Monthly household income (Euros)	N	1038
	< 1000	183 (17.6%)
	1000 – 1500	302 (29.1%)
	1500 – 2000	241 (23.2%)
	> 2000	312 (30.1%)
Financial support	Private health insurance	1013/1042 (97.2%)
	Coverage for long-lasting illness	769/1052 (73.1%)
	Personalised allowance of autonomy	325/1040 (31.3%)
Human support	At least one human support	973/1085 (89.8%)
	Relatives	582/910 (64.0%)
	Domestic help	502/949 (52.9%)
	Nurse	506/933 (54.2%)
	Home care nurse service	78/854 (9.1%)
	Physiotherapist	271/882 (30.7%)
	Meals-on-wheels	222/881 (25.2%)
	Housekeeper	153/871 (17.6%)
	Assistant social worker	106/853 (12.4%)
	Other	197/789 (25.0%)
Technical support	N	1083
	At least one technical help	908 (83.8%)
	Alarm system	664 (61.3%)
	Stick	467 (43.1%)
	Crutch	138 (12.7%)
	Walker	197 (18.2%)
	Grab bar	494 (45.6%)
	Chair wardrobe	173 (16.0%)
	Booster seat	161 (14.9%)
	Wheelchair	74 (6.8%)
	Medical bed	107 (9.9%)
	Lifting apparatus	4 (0.4%)
	Other	455 (42.0%)

Overall, 68.0% were living in a house. Most dwellings appeared not fully adapted including presence of onside or outside stairs (78.2% and 58.7%, respectively); 50.2% had a shower and 39% a bathtub but in most cases, they were not adapted, and/or not accessible(See Supplementary Table S2).

### Geriatric interventions

At the end of the assessment, it was considered that 381/1063 (35.8%) subjects could stay at home without difficulties, while 637 (59.9%) subjects required some help. Staying at home was considered as only possible in condition of human support in 681 (64.0%) subjects, technical support in 503 (47.3%) subjects, or financial support in 409 (38.5%) subjects. Institutionalisation was requested for 59 (5.5%) subjects and should be considered in 149 (14.0%) other subjects. A description of proposed interventions following the geriatric assessment is provided in Table 3. This included preventive therapeutic and nutritional advices (78.2% of subjects), a medical consultation (68.2%), an evaluation by a social worker (42.1%), or a psychomotor therapist (42.1%), and therapeutic modification (39.8%).

**Table 3:** Geriatric interventions proposed at the end of first visit

Medical plan	740 (68.2%)
Preventive hygieno-therapeutic advices	848 (78.2%)
Consultation (family physician, specialist)	741 (68.2%)
Evaluation by a social worker	457 (42.1%)
Evaluation by a psychomotor therapist	457 (42.1%)
Therapeutic modification	432 (39.8%)
Evaluation by an occupational therapist	328 (30.2%)
Complementary exam	170 (15.7%)
Scheduled hospitalisation	125 (11.5%)

## Discussion

The goal of this preliminary report was to determine the profile of the FREEDOM-LNA cohort. The cohort included 1085 community-dwelling elderly subjects, in advanced age (83 years on average) and composed of women in majority. More than half of subjects were living alone, had several cardiovascular risk factors and are exposed to polypharmacy. Overall, they presented with a very low physical capacity and mobility-related disability and 30-50% showed significant cognitive deficit, depression and a risk of malnutrition. Most subjects were frail or prefrail and in loss of independence and thus required help to stay at home. Overall, the health status based on the EQ-5D questionnaire was worse compared to another cross-sectional study in advanced elderly, [33] with a substantial proportion of subjects reporting pain/discomfort, mobility difficulties, and anxiety/depression. The overall health status of the FREEDOM-LNA population was also worse compared to a cohort of community-dwelling older subjects selected for their ability to walk 20 feet without personal assistance, [34] but quite better compared to another small clinical trial in our clinical centre with frail elderly people, [35] and compared to older adults admitted to our emergency geriatric medicine unit [36].

Disability in essential daily activities is considered as an adverse outcome of frailty. In this study, 32% of subject were frail based on the Fried’s criteria and another considerable proportion (58%) was pre-frail. In a systematic review of the literature, the reported prevalence of frailty in elderly among the community worldwide was variable ranging between 4% and 59% and the meta-analysis showed a weighted prevalence of 10.7%, [37] which is quite lower compared to the rate in the FREEDOM-LNA study. In another literature review, Shamliyan et al. estimated the prevalence of the frail phenotype to 26% in people over 85 years [38]. In another cross sectional study in France, physical frailty was reported in 9.5% of people aged 70-79 years, 18.4% of people 80-89 years, and 25.3% of people aged ≥ 90 years [39]. In our study, the most frequent frailty criteria were weakness and low activity. These two frailty criteria have been shown to be the most powerful predictors of ADL disability [40]. In this study, it is noteworthy that the rate of subjects with some disabilities was substantially higher (66.9% had difficulties in at least one ADL, and 85.2% in at least one IADL) than the rate of frail subjects. By comparison, in another French cross-sectional study, 15.0 and 22.4% of elderly of similar age had difficulties in at least one ADL and IADL, respectively [41].

Loss of independence in our cohort was consistent for more than 51.3% of subjects as indicated by the GIR scores, and 59.5% of subjects using SMAF. They predominantly needed help to do most executive functions and for “grooming”. Other daily activities were performed with difficulties, and some may be limited to alteration in mobility (“walking outside”, or “using stairs) or mental function (“memory”, “judgement”). Overall, the disability profile is consistent with an early phase of loss of independence [42]. Possible causes of loss of independence included musculoskeletal and neurological disorders which were reported by 56 and 32% of subjects, respectively, and also cognitive or mental decline in 30-50% of subjects. Our results also showed that almost 75% had a risk of mobility-related disability as assessed using the SPPB questionnaire. A low SPPB score has been previously associated with an increased risk of frailty, disability in daily life activities, falling, hospitalisation, and nursing home admission. [43, 44]. Moreover, social isolation and depression can also lead to frailty and decline in functional status [45]. In this cohort, more than half of the population were living alone and 49% had signs of depression. On the other hand, the home environment can also influence the ability to perform ADL, and we found that it was frequently not adapted with stairs and inaccessible showers or bathtubs.

Nutrition is believed to influence age-related frailty, cognition and disability, and adverse health outcome [46, 47]. Here, we used the MNA questionnaire which can be considered as a valuable tool to identify frail elderly subjects at risk of malnutrition, especially because it encompasses physical and mental aspect of health including mobility, psychological stress or acute disease in the previous 3 months [20]. It can also predict the risk of malnutrition when serum albumin and BMI are still normal, which was the case in the FREEDOM-LNA cohort. Here, we found that 7.5% of subjects were clearly malnourished (MNA < 17) which seems low compared to the rate of frailty subjects and compared to another small clinical trial in frail older subjects referred to our clinical centre [35]. Nevertheless, a high proportion (37%) of subjects is considered at risk of malnutrition.

Taken together, the baseline characteristics of the FREEDOM-LNA cohort showed a heterogeneous population of elderly particularly aged, frail or prefrail and presenting with frequent multimorbidity, and at risk of loss of independence due to low physical capacity and alteration of cognition. At the end of this geriatric assessment, it was considered that most subjects needed human support to be able to stay at home. Technical and financial conditions may be an issue, thus requiring intervention. The independent factors associated with frailty, functional loss and cognition will be analysed in an upcoming report.

As observational, our cohort has some limitations, mainly due to selective and information biases. First, the cohort was composed of community-dwelling subjects who were interested to receive a comprehensive geriatric assessment at home. Thus, such assessment may be less considered in apparently healthy elderly subjects. In addition, it is not known exactly if the subjects were addressed for primary or secondary prevention. According to an estimation between 2010 and 2017, interventions by our clinical centre were mainly solicited by the subject or a relative (45.5%), followed by hospital (30.8%), familial physicians (15.4%) or others (7.9%) (Personal data, not published). Nevertheless, our aim was not to obtain a representative sample of the general population, but rather to constitute an observatory of elderly subjects at risk of loss of independence. Next, we used the frailty criteria defined by Fried et al [10]. This is the most frequent screening tool used for frailty and was shown to be independently predictive of incident falls, worsening mobility or ADL disability, hospitalization, and death in the elderly. However, this restricts the multidomain of frailty to a physical phenotype, and thus do not completely consider the impact of cognitive and emotional function in development and progression of frailty [48]. Nevertheless, various neuropsychological tests were used in our study to measure cognitive and depressive functions and their relationship with disability and frailty. This will be analysed in separate reports. Finally, some percentages may be overestimated due to missing data including cognitive tests (i.e. GDS, CDT, verbal fluency) and frailty.

In conclusion, the FREEDOM-LNA cohort is composed of advanced elderly with various risk factors of frailty and disability associated with low health status, and impaired physical and cognitive functions. This cohort will help to determine factors that adversely influence the trajectory of physical frailty over time.

## Supplementary Information

**Additional file 1: Table S1.** EQ-5Dquestionnaire. **Table S2.** Habitation and equipment.

## Data Availability

Doctor Sophie Boyer, PhD (sophie.boyer@chu-limoges.fr) who should be contacted if someone wants to request the data. Data are not publicly available due to privacy or ethical restrictions.

## Abbreviations

ADL: Activity of Daily Living
AGGIR: Instrument for evaluating dependency in elderly in France
CGA: Comprehensive Geriatric Assessment
EQ-5D: EuroQol-5 Dimension
FRIED’S CRITERIA: Physical frailty
FREEDOM-LNA cohort: French acronym for Frailty, Clinical Research and Evaluation at Home in Limousin – Nouvelle Aquitaine
GDS: Geriatric Depression Scale
IADL: Instrumental Activities for Daily Living
MMSE: Mini Mental State Examination
MNA: Mini Nutritional Assessment
SMAF: Functional Autonomy Measurement system
SPPB: Short Physical Performance Battery
UPSAV: Geriatric mobile team (*Unité de Prévention de Suivi et d’Analyse du Vieillissement*)
