# Supplementary material for: Frailty and functional dependence in older population: lessons from the FREEDOM Limousin – Nouvelle Aquitaine Cohort Study
Source: BMC Geriatr. 2022 Feb 14;22:128. doi: 10.1186/s12877-022-02834-w (PMC8845396; doi:10.1186/s12877-022-02834-w)
Supplement: Supplementary file 1 — Additional file 1: Table S1. EQ-5Dquestionnaire. Table S2. Habitation and equipment. [file 12877_2022_2834_MOESM1_ESM.docx]

**Table 1S: EQ-5D questionnaire**

|  | | **Total N=1085** |
| --- | --- | --- |
| **Mobility** | N | 859 |
|  | Missing | 226 |
|  | I have no problems in walking about | 366 (42.6%) |
|  | I have some problems in walking about | 480 (55.9%) |
|  | I am confined to bed | 13 (1.5%) |
|  | | |
| **Self-care** | N | 859 |
|  | Missing | 226 |
|  | I have no problems with self-care | 493 (57.4%) |
|  | I have some problems washing or dressing myself | 328 (38.2%) |
|  | I am unable to wash or dress myself | 38 (4.4%) |
|  | | |
| **Usual activities** (e.g. work, study, housework, family or leisure activities) | |  |
|  | N | 859 |
|  | Missing | 226 |
|  | I have no problems with performing my usual activities | 328 (38.2%) |
|  | I have some problems with performing my usual activities | 456 (53.1%) |
|  | I am unable to perform my usual activities | 75 (8.7%) |
|  | | |
| **Pain/ discomfort** | |  |
|  | N | 859 |
|  | Missing | 226 |
|  | I have no pain or discomfort | 176 (20.5%) |
|  | I have moderate pain or discomfort | 603 (70.2%) |
|  | I have extreme pain or discomfort | 80 (9.3%) |
|  | | |
| **Anxiety/ depression** | |  |
|  | N | 859 |
|  | Missing | 226 |
|  | I am not anxious or depressed | 280 (32.6%) |
|  | I am moderately anxious or depressed | 503 (58.6%) |
|  | I am extremely anxious or depressed | 76 (8.8%) |
|  | | |

**Table 2S: Habitation and equipment**

| Urban  Rural | 721/1083 (66.6%)  362/1083 (33.4%) |
| --- | --- |
| House  Apartment  Other | 737/1083 (68.1%)  313/1083 (28.9%)  33/1083 (3.0%) |
| Presence of a floor  Outside stairs  Elevator  Inside stairs  Small spaces  Large spaces  Shower  Adapted  Accessible  Walk-in  Bathtub  Adapted  Accessible  Walk-in | 910/1075 (84.7%)  625/1070 (58.4%)  224/1071 (20.9%)  836/1070 (78.1%)  281/1063 (26.4%)  826/1074 (76.9%)  544/1083 (50.2%)  148/544 (27.2%)  97/544 (17.8%)  160/544 (29.4%)  422/1083 (39.0%)  28/422 (6.6%)  24/422 (5.7%)  32/422 (7.6%) |
